# Gabapentin Treatment for Neuropathic Pain in a Child with Sciatic Nerve Injury

**DOI:** 10.1155/2015/873157

**Published:** 2015-08-04

**Authors:** Halil Ekrem Akkurt, Haluk Gümüş, Hamit Göksu, Ömer Faruk Odabaşı, Halim Yılmaz

**Affiliations:** ^1^Department of Physical Medicine and Rehabilitation, Konya Training and Research Hospital, 42090 Konya, Turkey; ^2^Department of Neurology, Manisa State Hospital, 45020 Manisa, Turkey

## Abstract

There are a restricted number of studies about usage of gabapentin for neuropathic pain treatment of pediatric patients. We shared a 12-year-old male case with severe neuropathic pain that hindered the rehabilitation programme for the loss of muscle power and movement limitation. Neuropathic pain developed after peripheral sciatic damage due to firearm traumatisation did not respond to other medical treatments but healed nearly completely after gabapentin usage.

## 1. Introduction

Sciatic nerve is the largest and the longest peripheral nerve in human body. Surgical interventions, prosthesis operations, fractures, dislocations, contusions of hip joint, firearm traumatism, compression syndromes, and intramuscular gluteal injections may cause sciatic nerve injuries.

For high level sciatic nerve lesions, motor signs like loss of muscle strength for knee flexion, foot dorsiflexion, and plantar flexion could be found, as well as paresthetic symptoms including burning and knife-like pain along sciatic nerve. Neuropathic pain symptoms like hyperalgesia and allodynia and trophic changes including cold extremities, erythema, thinning of skin, changes on nails, vasomotor changes, and loss of sense in posterior of thigh, lower half of the leg, and foot may be seen [1–3].

Neuropathic pain is defined as a kind of pain related to the injury and/or dysfunction of peripheral or central nervous system or alterations in the stimulations of these structures. The pathophysiology of neuropathic pain is so complicated that it is not fully understood [4].

Neuropathic pain may develop because of sciatic nerve injury. There are some guidelines for neuropathic pain that developed due to sciatic nerve injury or various nerve injuries treatment in adults. Although the effectiveness of gabapentin in neuropathic pain treatment was shown in various studies, it was reported that there are only a few studies about gabapentin usage in children [5]. Also till today, there have been restricted literature information about the usage of gabapentin on pediatric patient who has neuropathic pain after sciatic nerve injury [6].

In this case report, we discussed a 12-year-old male case with severe neuropathic pain that developed after peripheral sciatic damage due to firearm traumatisation and hindered the rehabilitation programme planned to treat the loss of muscle strength and limitation of movement caused by peripheral sciatic damage. The pain did not respond to other medical treatments but healed nearly completely after gabapentin treatment.

## 2. Case

A 12-year-old boy was brought to our policlinic by his family with a complaint about weakness in his right leg and inability to walk. His family told us that it was caused by the firearm injury which had occurred two months before.

On physical examination, it was found that the patient had incision scars left as a result of surgical intervention made two months before to take out the buck shots in medial and posterior regions of the thigh after the firearm injury. It was written in his surgical report that he had sciatic nerve injury and the buck shots in the body had been taken out. Sense and reflex examination could not be made because of strong pain. The intensity of pain was measured by visual analogue scale (VAS) ranging from 0 to 10 and values were 10 and on a scale for assessment of neuropathic symptoms and signs (LANSS) were 22 before treatment. Muscle-power examination could not be made, either, because of the strong pain. However, as far as it was evaluated there was no active movement in right foot: dorsiflexors and plantar-flexors. In evaluating the range of motion, a goniometric measurement was taken with minimal contact to the patient due to severe pain, and the right knee was measured in 105-degree limited flexion (75-degree extension was needed for complete extension) and the right foot ankle in 20-degree limited plantar flexion (20 degrees was needed for neuter position of foot ankle). The patient was accepted to our clinic for rehabilitation. As it is planned to give medicine to the patient, it is aimed to determine whether the values in liver and kidney were normal and there was infection and diabetes or not, so the blood analyses of the patient were asked. AST, ALT, fast blood, glucose, creatine, hemogram sedimentation, and C-reactive protein (CRP) tests were found to be normal.

There was no pathologic substrate in X-rays of right knee and ankle image explaining joint movement limitation and severe pain. Electroneuromyography examination on the patient could not be evaluated definitely because of the pain; however, it was determined that there was nearly a total partial axonal degeneration which was heavier in the right sciatic nerve peroneal division and lighter in tibial division. Soft tissue ultrasound of the gluteal region; venous and arterial doppler ultrasound of right lower extremity, were normal.

As the patient had to keep his knee in flexion position due to severe pain and movement limitation, and his X-ray and ultrasound images were normal, CT and MRI were not asked. This pain in patient was evaluated as allodynia. A rehabilitation programme (stretching, increasing the range of motion, proprioception, strength exercises, TENS, and surface heaters) was planned for the patient but could not be applied because of severe allodynia. As the rehabilitation programme could not be implemented, we consulted the paediatric neurologists and acted on their suggestions. Thus, topical and oral nonsteroidal anti-inflammatory medication according to the patient's age was given to the patient for ten days. As there was no decline in complaints and the rehabilitation programme could not be implemented, daily 10 mg/kg dose gabapentin was given to the patient by consulting paediatric neurology. Regarding patient complaints, the dose of medicine was increased up to 16 mg/kg a day. Beginning from the first week, the patient's neuropathic pain complaints declined; thus, a rehabilitation programme could be implemented. The patient's value of VAS and LANSS was 3 and 0, respectively, in the second week. Apart from light dizziness for the first 1-2 days, there were no side effects during gabapentin treatment. In the twentieth séance after the start of gabapentin treatment; flexion and extension of right knee, right foot ankle plantar flexion was unrestricted, and right ankle dorsiflexion was measured as 100 degrees. Foot plantar flexion and dorsiflexion were 3/5, and right knee flexion, extension, right hip flexion, extension, abduction, and abduction of muscle powers were 5/5 level. The patient was discharged and given home exercises programme. The medicine was lessened gradually to the end of the second month. The patient came for monthly control each month, and he did not have any additional complaints.

## 3. Conclusion

Sciatic nerve originates from L4-S3 spinal nerves. Passing through greater sciatic foramen under piriformis muscle, it parts from pelvis and goes together with inferior gluteal nerve and posterior femoral cutaneous nerve. Since sciatic nerve takes place around hip joint, the most common cause of sciatic neuropathies is traumas. In patients with sciatic neuropathic pain, the commonest complaint was weakness. This paralysis occurs in severe nerve lesions, within hamstring muscles and in all muscles under knee. Numbness or complaints like paresthesias are commonly seen on most patients, most of whom complain about dysesthetic pain in sciatic nerve trace [7–9].

Main approach in neuropathic pain treatment in children is like in adults [10]. Tricyclic antidepressants, serotonin-noradrenalin reuptake inhibitors, the agents (gabapentin and pregabalin) that bind to N type Ca+2 channels, and topical lidocaine as are the first step, and opioids are the second step medicines [11]. Gabapentin, an anticonvulsant agent, which was asserted to reduce presynaptic glutamate release and, binding to postsynaptic calcium channel, prevent central desensitization due to glutamate neurotransmission in the dorsal horn of the spinal canal, was used in neuropathic pains because of its tolerability and good effect [12]. Although the effectiveness of gabapentin in neuropathic pain treatment on adults was shown in various studies, it was reported that there are a few studies about gabapentin usage in children. On a few studies, gabapentin was used on children with neuropathic pain developed after thoracotomy, complex regional pain syndrome, or cerebral palsy, and it was found highly effective [5, 13, 14]. Gabapentin was used for multimodal analgesia in paediatric patients having neuropathic pain due to cancer. Butkovic et al. have reported that it could be safely used with minimal side effects [15]. The aim of neuropathic pain treatment is not only to decrease pain but also to improve functionality and life quality. Gabapentin is considered to be at the beginning of the list to improve the patients' quality of life [16]. Although gabapentin is more expensive than amitriptyline, the fact that it has fewer side effects and is more tolerable, it is an important advantage for its use in children [5]. In order to lessen side effects, it should be started with a minimum dose and, considering its effectiveness in pain treatment, be increased gradually [5, 17]. It was reported that 26–78 mg/kg gabapentin daily dose was safely used in children [18]. The most common side effects of gabapentin treatment are dizziness, somnolence, weakness, and peripheral edema [11].

As our 12-year-old patient did not respond to other medical treatments, and we could not apply our intended rehabilitation programme, we started giving a 10 mg/kg/day dose. Regarding the patient's complaints, we increased gabapentin dose up to 16 mg/kg/day in two weeks after consulting paediatric neurology department and referring to the data in the literature. Apart from a light dizziness present for the first 1-2 days, there were no side effects during gabapentin treatment. There was considerable improvement in patient's neuropathic complaints, so we were able to start to apply the planned rehabilitation programme. This case shows that gabapentin treatment is effective with minimal side effects in treating severe neuropathic pain that hinders rehabilitation programme related to the loss of muscle strength and the limitation of range of joint motion developed after peripheric sciatic nerve injury. In order to be able to speak more certainly, we think that further studies are needed.
